# International comparison of professional competency frameworks for nurses: a document analysis

**DOI:** 10.1186/s12912-023-01514-3

**Published:** 2023-09-28

**Authors:** Renate F. Wit, Anke J.E. de Veer, Ronald S. Batenburg, Anneke L. Francke

**Affiliations:** 1 Netherlands Institute for Health Services Research (Nivel), PO Box 1568, Utrecht, 3513CR The Netherlands; 2 Department of Sociology, Radboud University Nijmegen, Nijmegen, 6525 XZ The Netherlands; 3 Department of Public and Occupational Health, Amsterdam Public Health Research Institute, Amsterdam University Medical Centre and Vrije Universiteit Amsterdam, Van der Boechorststraat 7, Amsterdam, 1081BT The Netherlands

**Keywords:** Nursing, Competencies, Professional Competency Frameworks, Registered nurse

## Abstract

**Background:**

Nursing competency frameworks describe the competencies; knowledge, skills and attitudes nurses should possess. Countries have their own framework. Knowledge of the content of professional competency frameworks in different countries can enhance the development of these frameworks and international collaborations.

**Objective:**

This study examines how competencies and task divisions are described in the current professional competency frameworks for registered nurses (RNs with a Bachelor’s degree) in the Netherlands, Belgium, the United Kingdom (UK), Canada and the United States (US).

**Methods:**

Qualitative document analysis was conducted using the most recently published professional competency frameworks for registered nurses in the above-mentioned five countries.

**Results:**

All the competency frameworks distinguished categories of competencies. Three of the five frameworks explicitly mentioned the basis for the categorization: an adaptation of the CanMEDS model (Netherlands), European directives on the recognition of professional qualifications (Belgium) and an adapted inter-professional framework (US). Although there was variation in how competencies were grouped, we inductively identified ten generic competency domains: (1) Professional Attitude, (2) Clinical Care in Practice, (3) Communication and Collaboration, (4) Health Promotion and Prevention, (5) Organization and Planning of Care, (6) Leadership, (7) Quality and Safety of Care, (8) Training and (continuing) Education, (9) Technology and e-Health, (10) Support of Self-Management and Patient Empowerment. Country differences were found in some more specific competency descriptions. All frameworks described aspects related to the division of tasks between nurses on the one hand and physicians and other healthcare professionals on the other hand. However, these descriptions were rather limited and often imprecise.

**Conclusions:**

Although ten generic domains could be identified when analysing and comparing the competency frameworks, there are country differences in the categorizations and the details of the competencies described in the frameworks. These differences and the limited attention paid to the division of tasks might lead to cross-country differences in nursing practice and barriers to the international labour mobility of Bachelor-educated RNs.

**Supplementary Information:**

The online version contains supplementary material available at 10.1186/s12912-023-01514-3.

## Introduction

Since the late 19th century, the nursing profession in the Netherlands and many other Western countries, like the United Kingdom and the United States of America, has become a paid profession with specific nursing training [1, 2]. Nursing is not a static profession, but constantly evolving under the influence of external and internal factors, i.e. developments affecting the context of the profession and developments within the profession [3].

External factors that influence professional development include demographic and epidemiological developments. For instance, the increase in patients with chronic conditions and high-aged people with multimorbidity requires nurses to be able to provide complex care and support across multiple (physical, psychological and social) domains [4]. Also, healthcare policies increasingly focus on prevention and promoting a healthy lifestyle [5, 6]. Developments in ICT also have an impact: nurses increasingly use web information, electronic records and online communication [7–10]. In addition, much attention is paid in society and healthcare policy to the autonomy of the individual professional and their degree of control [11, 12]. Also in nursing practice, self-management support and the promotion of the autonomy of those in need of care has become increasingly important [13, 14].

Internal factors also influence the nursing profession. Internal factors concern, for instance, the continuing academic development in nursing. The number of university departments of nursing science is growing in Western countries, as is the number of nursing scholars; this is associated with the increased attention paid to evidence-based nursing practice [15, 16]. Another factor concerns the labour market; most countries in the Western world have shortages of skilled health workers [17, 18]. Together with the shortage of physicians, the increase in the specializations in nursing is leading to or enabling task shifts between physicians and nurses [19–21]. Task shifting is one aspect of the changing division of tasks, whereby the task is allocated in part or in full to another profession. Examples are task shifts from physicians to (specialized) nurses in the prescription of medication and the monitoring and treatment of people with chronic conditions [22, 23].

It can be expected that such internal and external factors, and their influence on the nursing profession, are reflected in the professional competency frameworks for nurses. Equally, up-to-date professional competency frameworks influence the nursing profession. In this paper, we define a professional competency framework as a document describing the competencies a nurse must have. ‘Competencies’ are the knowledge, skills and attitudes, and the ability to perform tasks successfully within the professional context [11, 24].

Professional competency frameworks are often developed and/or authorized by national professional nursing associations [11, 24]. There is no standard procedure or guide for developing a professional competency framework for healthcare professionals. However, an existing educational model, such as CanMEDS, might be used as the foundation for the description of competencies [11] as this is sometimes used for structuring nursing education curricula [25] and for example the professional competency framework in the Netherlands [26]. CanMEDS is the abbreviation of Canadian Medical Educational Directives for Specialists and describes roles such as Expert, Communicator, Collaborator, Leader, Health Advocate, Scholar and Professional [27]. We expected this model would be used as the point of departure in some of the professional competency frameworks of nurses in other Western countries.

Up-to-date professional competency frameworks are important as they provide guidance to nursing practice, but also reflect the required core competencies of the nursing profession in a specific country. Under the influence of internal and external factors, the content of the nursing profession evolves in a dynamic institutional process. This means that professional competency frameworks have to be revised regularly. For instance, in the Netherlands and Belgium, nursing associations are currently preparing a new competency framework for nurses. For the development of future professional competency frameworks, it is relevant to know the content of professional frameworks in other countries: what competencies and tasks performed by nursing staff do they describe? By comparing them, developers of professional competency frameworks in one country can learn from other countries.

This document analysis therefore describes the competencies and task divisions between nurses and other professionals in the current professional competency frameworks for Bachelor-educated registered nurses (RNs) in five countries: the Netherlands, Belgium, the United Kingdom (UK), Canada and the United States (US). First, the choice for the Netherlands is based on the motherland of the authors, and Belgium is chosen as an interesting neighbouring country that has similarities with the Netherlands culturally, geographically and in terms of its language (Dutch is spoken by over half the Belgian population). The three Anglo-Saxon countries (UK, Canada and US) were selected because they have strongly influenced the global development of the nursing profession. This is particularly the case for the UK which initiated modern nursing through the pioneering role of Florence Nightingale, which first spread to Canada and the US as two countries that are historically and culturally strongly related to the UK. In these three countries, the nursing profession progressed rapidly in the second half of the 20th century due to factors such as advanced academic education for nurses and federal funding and support for nursing research [28, 29]. The five selected countries have a strong international orientation in common and have been previously analysed in a literature study into the roles and positions of nursing staff [30].

The following research questions were answered:

1. In each country, what is the educational model and/or base for the categorizations of the key competencies described in the professional competency frameworks for registered nurses (Bachelor educational level)?
2. What are the competencies described in the professional competency frameworks, and how do these differ or agree between the countries analysed?
3. How do the professional competency frameworks address the division of tasks between nurses and other healthcare professionals?

In addressing these research questions, we chose to focus on nurses with a Bachelor of Science/in Nursing, because that is the level of education that is most comparable between countries [30].

## Methods

### Searches and inclusion criteria

We performed a qualitative document analysis, a systematic procedure for reviewing or evaluating documents [31], of professional competency frameworks. To be included in this document analysis, documents had to:

- be a national professional competency framework for registered nurses with a Bachelor of Science in Nursing. If there was no separate competency framework for Bachelor-educated nurses, the framework for registered nurses in general was eligible for inclusion.
- be from the Netherlands (NL), Belgium (BE), Canada (CA), the United Kingdom (UK) or the United States (US).
- concern a general competency framework covering nursing in various healthcare sectors and various patient groups.
- be developed by or in collaboration with a national or international nursing association.
- be published in Dutch or English.
- be the most recent competency framework.

Documents focusing solely on education or nursing specializations (e.g. intensive-care nursing) or specific settings (e.g. community nursing) were excluded.

We identified five relevant professional competency frameworks (one for each selected country) between November 2021 and March 2022 through website searches of professional nursing associations and government sites. Our search for nationwide professional frameworks and nursing associations revealed only one nationwide general professional competency framework for registered nurses with a bachelor’s degree in each respective country.

RW and AF independently reviewed the documents against the inclusion criteria. Then the documents were checked to see whether they were the most recent versions by contacting experts (representatives of professional nursing associations and professors in Nursing) in the different countries.

### Analysis

The competency frameworks were analysed using a qualitative document analysis [31]. All competency frameworks were read thoroughly and repeatedly to become familiar with their content and to identify which educational model or other base the competency frameworks had used to categorize competencies in specific domains. Relevant fragments about competencies and competency domains were open-coded inductively. By performing constant comparisons of codes within and between the competency frameworks, and by grouping similar codes, we identified ‘generic’ competency domains that were addressed in all frameworks. The identified domains and competencies were discussed on various occasions within the research team (RW, AF, RB and AV), and the researchers returned to the texts of the competency frameworks several times to ensure that the results were grounded in the texts of the competency frameworks. This analysis process was performed in a similar way and partly in parallel to address the research question on task division. The coding and analysis process was supported by the use of MAXQDA 2022 [32].

To ensure the internal validity of the findings, both the pre-definitive results section and the schematic overview of the results (in Appendix 1) were presented for verification to experts from the respective countries. The pre-definitive results and the schematic overview were verified by country experts for the Netherlands, Belgium and the UK. The country experts we contacted for the frameworks of Canada and the US did not use the opportunity to verify our results.

## Results

### General characteristics of professional competency frameworks

Five professional competency frameworks were included; an overview of the included frameworks can be found in the Declarations under the section Availability of data and materials. The competency framework for the Netherlands (2015) was developed in collaboration with the Nursing Association in the Netherlands (V&VN) [26]. This country has competency frameworks for different educational levels of nurses bundled in one document. For this document analysis, only the competencies described for registered nurses at the Bachelor level have been analysed.

In Belgium (BE), in addition to higher education, there is also vocational training available for nurses. However, the existing framework does not have a separate framework or distinct section for nurses holding a Bachelor’s degree. The Belgian competency framework was published in 2016 and authorized by the Federal Council for Nursing [33]. This framework was published in Dutch and French. We used the Dutch version.

The frameworks for the UK and Canada were both published in 2018, by the Nursing & Midwifery Council (NMC) and the Canadian Council of Registered Nurse Regulators (CCRNr) respectively [34, 35]. In both countries, the competency frameworks only focussed on nurses with a Bachelor’s degree.

The US framework was published in 2021 by the American Association of Colleges of Nursing (AACN) [36]. It consisted of competencies at two levels: (1) entry-level competencies for registered nurses; and (2) advanced-level competencies (for advanced nursing practice). For this study the entry-level competencies were analysed; they concern the entry level for RNs with a Bachelor’s degree in Nursing [37].

### Basis for the categorizations

Three of the five professional competency frameworks mentioned explicitly what their point of departure was for the categorization of the competencies into specific domains: an adaptation of the CanMEDS model (NL), European directives on the recognition of professional qualifications (BE) and an adapted inter-professional framework (US).

In the UK competency framework, no reference was provided concerning the origin of the categories of the competencies. The Canadian framework does not mention the point of departure of the categorization of competencies either, but the categorization looks similar to the domains of the CanMEDS model.

### Ten identified generic competency domains for RNs

Although the original categorization and description of competencies differed between the countries, through the document analysis (see Methods) we were able to extract and distinguish ten ‘generic’ domains of competencies that are addressed in all competency frameworks (see Appendix 1). The ten generic domains were:

1. Professional Attitude;
2. Clinical Care in Practice;
3. Communication and Collaboration;
4. Health Promotion and Prevention;
5. Organization and Planning of Care;
6. Leadership;
7. Quality and Safety of Care;
8. Training and (continuing) Education;
9. Technology and e-Health;
10. Support of Self-Management and Patient Empowerment.

As can also be seen in Appendix 1, four of the ten generic domains have a similar title in the frameworks. These are the generic domains ‘Professional Attitude’, ‘Clinical Care in Practice’, ‘Health Promotion and Prevention’ and ‘Organization and Planning of Care’. Regarding the other six generic domains, there is more variation in the headings used in the competency frameworks.

Furthermore, the generic domain ‘Support of Self-Management and Patient Empowerment’ is not described in any framework as a separate domain (with a similar title), although all competency frameworks present this as a core element of nursing and pay substantial attention to the need to support the self-management and empowerment of patients.

All competencies described in the professional competency frameworks fit in one or other of the ten generic domains.

### Competencies within the generic domains

#### Professional attitude

The first generic domain concerns Professional Attitude. This is defined in our study as the willingness and ability to act professionally as a nurse complying with laws and regulations, evidence-based knowledge and ethical standards. This domain can be found as a separate domain in all frameworks, with the term ‘professional’ in the heading (see Appendix 1). The US competency framework names this domain ‘Professionalism’, although the content of the domain is largely similar to that of the other countries, which use the heading ‘Professional attitude’ for this domain. For all countries, the main competencies falling under this domain were ‘Ethical practice’, ‘Comply with laws, policies and regulations’, ‘Self-reflection’ and, for the UK and the US, also ‘Person-centred care’. For all countries, ‘accountability’ was also a core aspect of Professional Attitude.

#### Clinical care in practice

The generic competency domain Clinical Care in Practice is described in roughly the same way in all competency frameworks, as providing safe, evidence-based care while engaging with the patient in a caring relationship. This domain comprises competencies both for basic nursing care and for medical-technical care. As can also be seen in Appendix 1, it is a separate domain in all frameworks but with varying headings: ‘Care provider’ (NL), ‘Independently make a nursing diagnosis using current theoretical and clinical knowledge for the necessary nursing care’ (BE), ‘Providing and evaluating care’ (UK), ‘Clinician’ (CA) and ‘Person-centred care’ (US).

The Dutch competency framework described competencies such as gathering information in various ways about the person requiring care and at a more generic level; analysing, interpreting and applying this information; entering into a caring relationship; and carrying out restricted and high-risk actions. The Belgian framework emphasises the ability of RNs to independently make nursing diagnoses using evidence-based assessment techniques and deliver nursing care. In the UK, there is a focus on providing evidence-based compassionate and safe interventions, demonstrating knowledge to respond proactively and demonstrating the ability to provide nursing intervention and support. In the Canadian competency framework, RNs provide safe, competent, ethical, compassionate and evidence-informed care. The US framework describes competencies such as person-centred care focusing on the individual within multiple complicated contexts, delivering regenerative or restorative care and establishing caring relationships.

#### Communication and collaboration

The generic competency domain Communication and Collaboration is described in four of the frameworks under two separate competency headings. The exception is the US, where it is described under one main heading and where individual competencies that refer to communication and collaboration are found under several of the framework headings. As communication and collaboration are closely related, we have grouped them into one domain.

In the Dutch competency framework, the relevant competencies are described under ‘Communicator’ (respectful and proficient, verbal, nonverbal and digital communication) and ‘Collaborator’ (working with patients, their network, professionals, multidisciplinary teams, and efficient and effective reporting). The Belgian framework refers to competencies regarding communication and collaboration under ‘Communicating professionally with clarity’ (active listening, emphasizing, respecting opinions and reporting and sharing information) and ‘Cooperation’ (working together with patients, their network and interdisciplinary teams). Additionally, one specific competency was found under another heading, namely building a culture of collegiality, respect and professional relationships. In the UK framework, communication and collaboration competencies are grouped under ‘Leading and managing nursing care and working in teams’ (play an active and equal role in the interdisciplinary team) and ‘Coordinating care’ (apply the principles of partnership, collaboration and interagency). Furthermore, three competencies were identified in other domains. In the Canadian framework, competencies are described under ‘Communicator’ (create and maintain professional relationships, share information, foster therapeutic environments, engage in active listening and effective communication in complex situations, report clearly) and ‘Collaborator’ (play an integral role in the healthcare team, initiate collaboration and determine their own professional and inter-professional role). In the US competency framework, RNs’ communication and collaboration competencies are grouped under ‘Inter-professional partnerships’ (intentional collaborations with care team members, patients, communities and other stakeholders to optimize care), while the competencies ‘communicating effectively with individuals’ and ‘promoting collaboration by clarifying responsibilities’ are described under other headings.

#### Health promotion and prevention

The generic competency domain Health Promotion and Prevention concerns health promotion and prevention by RNs, both directed at individual persons and public health in general. It is described in all frameworks as a separate domain, but under varying headings (see also Appendix 1).

For the Netherlands, this is described under the heading of ‘Health Promotor’ with competencies like carrying out interventions, collective prevention and health education, and providing input for policy-makers. The Belgian competency framework requires RNs to promote patient health and a healthy environment under ‘Empowering individuals, families and groups to adopt healthy lifestyles and care for themselves’. This involves providing information and teaching behaviour change strategies to the patient. For the UK, competencies are described under ‘Promoting health and preventing ill health’ and focus on improving and maintaining health and understanding and applying health promotion goals. In the Canadian framework, health promotion and prevention are discussed under ‘Advocate’. Additionally, two competencies under another heading align with Health Promotion and Prevention: use strategies to promote wellness, prevent illness, and to minimize disease and injury in clients, self, and others; and implement evidence-informed practices for infection prevention and control. In the US competency framework, relevant competencies under ‘Population Health’, include disease management of populations and evidence-based patient teaching materials.

#### Organization and planning of care

The generic competency domain Organization and Planning of Care has two aspects: the domain encompasses competencies regarding the planning of care and competencies regarding the system for organizing care, both within the institution and together with other healthcare institutions. It is described as a separate domain in all five frameworks, but individual competencies are also described under other headings.

In the Dutch competency framework, competencies related to organization and planning of care are mainly described under ‘Organizer’. These include being able to make decisions about policy, and coordinating and evaluating patient care. For Belgium, relevant competencies are described under ‘Managing the care process’, which involves utilizing available resources efficiently and planning nursing care. In the UK, organizational competencies are primarily described under ‘Assessing needs and planning care’ including developing person-centred care plans, understanding the mechanism to influence organizational change, and coordinating, leading and managing the needs of people. In the Canadian framework, competencies are described under the role of ‘Coordinator’. RNs should help clients to navigate healthcare systems, develop care plans, consider organizational culture and use resources wisely. Under the heading ‘Systems-based practice’, the US competency framework describes competencies for coordinating resources, applying knowledge of systems to work effectively, developing a care plan and organizing and coordinating care.

#### Leadership

The generic competency domain Leadership is addressed differently across the countries. Competencies regarding leadership are described under various headings in the Dutch and Belgian competency frameworks, while in the other three competency frameworks they are described as a separate domain (see also Appendix 1).

In the Dutch competency framework, relevant competencies are described under the headings: ‘Professional and personal leadership’, ‘Perform the job confidently and assertively’ and ‘Fulfil a coordinating role within a multidisciplinary team’. The relevant competencies described in the Belgian competency framework, are nursing leadership (taking the initiative in coordinating care), demonstrating professional leadership by participating in activities aimed at guiding policy and health services, making services more accessible, and organizational leadership. These competencies are described in different parts of the competency framework. In the UK competency framework, relevant competencies are described under the heading ‘Leading and managing care and working in teams’. The competency framework describes RNs’ leadership competencies in coordinating the care, acting as a role model and understanding the principles of effective leadership. Under the heading ‘Leader’, the Canadian competency framework describes RNs’ role as leaders who influence and inspire others, enhance the quality of a professional and safe practice environment and demonstrate self-awareness. In the US competency framework, competencies are grouped under the heading ‘Personal, professional and leadership development’. The competencies in this section include demonstrating commitment to personal health and wellbeing, showing professional maturity and developing leadership capacity.

#### Quality and safety of care

The generic domain Quality and Safety of Care is described as a separate domain for all countries except Canada, where it is covered extensively under other headings (see also Appendix 1). This generic domain concerns competencies regarding evidence-based practice, evaluation and documentation, assessing risks to safety and enhancing quality of care. All competency frameworks describe the competencies required for safe and high-quality care, e.g. competencies for evidence-based care, evaluation of care and improving the quality of care. The five competency frameworks use slightly different terms and differ in whether they focus on no harm (Canada and the US) rather than safe care (Netherlands, Belgium, and the UK).

More specifically, the Dutch competency framework, competencies regarding quality and safety of care are described under the heading ‘Professional and quality promotor’. In the Belgian competency framework, the competencies are described under ‘Analyse, evaluate and ensure the quality of care provision in order to improve one’s practice’, and in the UK under ‘Improving safety and quality of care’. As said, in the Canadian competency framework the quality and safety of care are not described in a separate domain. Competencies are listed in various parts of the competency framework, under the headings ‘Clinician’, ‘Professional’ and ‘Advocate’. In the US framework, the relevant competencies are mainly described under ‘Quality and Safety’.

#### Training and (continuing) education

The generic domain Training and (continuing) Education encompasses lifelong learning for nurses and providing or assisting in education. It is a separate domain in Canada and the US, but is described in the frameworks of the other three countries as well (see Appendix 1). Competencies regarding Training and (continuing) Education are described most extensively in the Netherlands, Belgium and the UK.

In the Dutch competency framework, relevant competencies are addressed under different headings. These include supervising and coaching colleagues, keeping up with professional literature, self-reflection, giving and receiving feedback and acting as a role model. For Belgium, relevant competencies are described under various headings in the framework. It emphasizes that RNs should evaluate themselves and sharpen their competencies through training and participating in research and the education of students and colleagues. In the UK competency framework, competencies for training and continuing education are described under various headings. This includes self-reflection and professional skill development, supporting and supervising students and providing constructive feedback. In Canada, competencies are described under ‘Educator’ and ‘Scholar’. ‘Educator’ includes competencies in selecting, developing and using relevant teaching and learning theories and strategies for diverse clients and contexts. ‘Scholar’ encompasses lifelong learning commitment, supporting research activities and developing research skills. In the US framework, competencies under ‘Scholarship for nursing discipline’, concern generating, synthesizing, translating, applying and disseminating nursing knowledge to improve health and transform care. Other competencies are described under different headings including educating individuals and families, engaging in peer evaluation and self-reflection, and identifying role models and mentors to support professional growth.

#### Technology and e-Health

The generic domain Technology and e-Health addresses competencies for digital literacy and the professional use of e-health. Only in the US is this described under a separate heading (‘Informatics and healthcare technologies’). In the other countries it is covered throughout the framework under various headings.

In the Netherlands, competencies include digital literacy, reporting digitally and working with electronic patient files, utilizing social media, remote care and e-health technologies. For Belgium, relevant competencies concern using technology and ICT to store, access and record data for improved healthcare access and patient outcomes. The framework refers to clear digital communication and digital literacy for RNs. According to the UK competency framework, RNs need numeracy, literacy, digital and technological skills to meet the needs of people in their care and to ensure safe and effective nursing practice. They should be able to utilize digital technologies for accessing information, recording vital signs, and interpreting data. The Canadian competency framework refers to using social media and ICT to uphold public trust in nurses. It also includes competencies for ICT communication, assisting patients with ICT, strengthening nursing informatics and identifying and analysing technologies that may change. In the US, technology and e-health competencies are bundled under ‘Informatics and healthcare technologies’. This includes the competencies to gather (digital) data, describe information and use communication technology tools for care delivery and documentation support.

#### Support of self-management and patient empowerment

The generic domain Support of Self-Management and Patient Empowerment is addressed in all the competency frameworks but not as a separate domain. The domain includes competencies aimed at enabling self-management by patients and empowering patients to take control of their health and to be involved in decisions about care and care interventions. In the Netherlands, Belgium and the UK, Support of Self-Management and Patient Empowerment is described in the introduction to the competency framework as a ‘core value’ of nursing. From that perspective, it is logical that the domain is not found under a separate heading but is discussed throughout the competency framework. References to self-management support and empowerment in Canada and the US are found under headings like: ‘Support and empower clients in making informed decisions about their health care’ and ‘Promote self-care management’.

### Division of tasks between RNs and other professionals

Professional competency frameworks not only refer to competencies, but also outline some tasks and may highlight aspects of the task division between RNs and other professionals. All five frameworks discuss at least one aspect of the division of tasks between RNs and other healthcare professionals. Most of the time, no details are given about healthcare professionals in terms of their discipline or whether they are other nursing staff with specific educational levels. Some competency frameworks mention delegating tasks from RNs to other healthcare professionals (Belgium, US) while others emphasize the coordination and organization of care or the delegation of tasks to other nursing staff (Netherlands). The UK framework mentions both delegating tasks and coordinating/organizing care. However, the specifics of task division and delegation are often unclear, with limited descriptions. For instance, one competency framework (Canada) only states that RNs must have knowledge of the delegation process. Information might partially overlap with the competencies described under the generic domain Communication and Collaboration.

In the Netherlands the division of tasks was described in the general description of the field of expertise of an RN with a Bachelor of Science in Nursing. This part describes the direction, organization, and coordination of the care process, including shared decision-making with the patient and other healthcare professionals, without specifying the disciplines of these other professionals. In addition, the Dutch competency framework states that for several restricted actions (in Dutch ‘*voorbehouden handelingen*’), the Bachelor-educated RN can have independent authority (in Dutch ‘*zelfstandige bevoegdheid*’), provided that the RN has the competency for the specific restricted action. If the RN has the required competency, the RN also has the authority to determine the indication for the restricted action, to perform the action herself, and to give orders to another healthcare professional (as referred to in Article 38 of the Individual Healthcare Professions Act) to perform the restricted procedure. Details are not given in the framework about who the other healthcare professionals can be, what the specific restricted actions with independent authority are or what the required competencies are. Regarding the independent authority there is a footnote stating that some medicines may be prescribed by specific groups of specialized nurses which is further explained in the appendix part of the document. In other restricted actions, the RN has functional independence (in Dutch ‘*functionele zelfstandigheid*’), which means that a competent RN can perform these without supervision but only after being instructed to do so by a doctor or other authorized healthcare professional. These restricted actions concern injections, catheterization of the bladder, insertion of a tube or drip and venipunctures.

In the Belgian framework, the task division between RNs and other professionals is described as delegating certain care aspects to an expert caregiver in an effectively and safely and accepting delegated activities that are in line with the RN’s competencies and legal professional field. Who these ‘expert caregivers’ are is not mentioned. The task division between RNs and physicians is described in a section about legal aspects. Nurses are allowed to contribute to the medical diagnosis by the physician, to execute the treatments prescribed by the physician and to carry out technical and nursing care actions for which a medical indication is not required. These tasks may be related to the physician’s diagnosis, the treatment prescribed by the physician, the administration of preventive medicines, and other medical actions that may be entrusted to a RN by a physician. The Belgian competency framework does not describe whether nurses have functional independence to perform actions related to medical treatments and if so, under what conditions. Neither does this professional competency framework mention whether physicians have full jurisdictional control (professional control) over all the actions of RNs related to medical treatments.

The competency framework for the UK describes task division firstly in relation to the RN’s competencies to safely and effectively lead and manage the nursing care of a group of people, demonstrating appropriate prioritization, delegation and assignment of care responsibilities to others involved in providing care. Secondly, task division is discussed in relation with the RN’s competency to show leadership as a role model in delivering high-quality nursing care. They are responsible for managing nursing care and are accountable for the appropriate delegation and supervision of care provided by team members and lay carers. RNs need to provide clear information and instructions when delegating or handing over care responsibilities. They should also demonstrate effective supervision, teaching, and performance appraisal by using clear instructions and explanations.

In the Canadian competency framework, task division is discussed is addressed under ‘Coordinator’ focussing on RNs’ competency in understanding the delegation process. No other references to task division were found in the professional competency framework. Moreover, this framework does not give any information on which tasks can be delegated and to which types of professional.

The US competency framework pays attention to the delegation of tasks, firstly in relation to RNs’ competency to delegate appropriately to team members and, secondly, their ability to delegate work based on team members’ roles and competencies. However, the framework does not specify the professional backgrounds of team members, which specific tasks can be delegated, or who has jurisdictional control over those tasks.

## Discussion

Three of the five professional competency frameworks included in this document analysis explicitly identify their point of departure in categorizing the competencies of RNs, namely the CanMEDS model (NL), European directives on the recognition of professional qualifications (BE) or an adapted inter-professional framework (US). The point of departure was not mentioned in the competency frameworks for the UK and Canada. In addition, one competency framework (BE) made no distinction between Bachelor-educated nurses and registered nurses with a vocational qualification.

Since there is no ‘golden standard’ for creating a professional competency framework for nurses, it is only to be expected that the categorizations and descriptions of competencies will differ between countries. However, variation in competency frameworks might be a barrier to the international exchange of the developing body of nursing knowledge and make it harder for nurses to work abroad. Within the European Union, there are minimum requirements for the training of general nurses in terms of content, placements, and time spent in clinical practice to facilitate the free movement of EU citizens [38, 39]. These are described in the EU directives 2005/36/EC and 2013/55/EU and have been used as a base for the Belgium professional competency framework [33, 38, 39]. Since European countries are required to incorporate these directives into nursing training and a specific guideline for implementation was provided by the European Federation of Nurses Associations (EFN) [40], we expected the EU directives were also referred to in the basis of the RNs professional competency frameworks in the Netherlands and UK, but this was not the case.

Around the world, shortages of skilled healthcare workers are high, but the need is more urgent in some countries than in others [41–43]. The further ageing of the population and the increase in complex care needs related to multimorbidity [4, 44] call for a strong and flexible health workforce in the future. The World Health Organization (WHO) recently published a roadmap to support countries and strengthen the nursing and midwifery professions in Europe [45]. In this roadmap, the WHO recommends aligning the competencies in different countries and creating good preconditions for international exchange. In addition, this roadmap discusses labour migration as a solution for country-specific workforce shortages.

Although the underlying model or base of the categorizations varied between countries, there were similarities in the competencies. We identified ten generic competency domains. These generic competency domains reflect the core of nursing and what is considered essential in today’s healthcare and in society in a broader sense. In particular the generic competency domains (4.) Health Promotion and Prevention, (9.) Technology and e-Health and (10.) Support of Self-Management and Patient Empowerment reflect the current focus of policymakers and practitioners on disease prevention, the use of ICT, and the promotion of patients’ self-reliance and autonomy. These developments are partly driven by the increasing shortages of nursing staff.

Although these three generic domains are addressed in all the included competency frameworks, there are also differences. Support of Self-Management and Patient Empowerment received less attention in the competency frameworks of Canada and the US than in frameworks for the Netherlands, Belgium, and the UK. Future international empirical research can show whether these differences are also found in daily nursing practice.

All professional competency frameworks address the division of tasks between nurses and other healthcare professionals but to a varying extent. Relatively most attention is paid to task division in the competency frameworks of the Netherlands and Belgium. These frameworks address the question of responsibility for medical actions carried out by the RNs, with both competency frameworks referring to legislation.

Only the Dutch and Belgian competency frameworks explicitly mention physicians regarding the division of tasks or task delegation, although all competency frameworks mention ‘other professionals’ in general when discussing task divisions or task delegation. The lack of clarity and explicitness regarding the specific tasks involved in the division of tasks is striking, especially as we have indications from previous research that task shifts, e.g. when prescribing medication or monitoring chronic conditions, occur increasingly in nursing practice [46]. This is also surprising because clarity and explicitness about which tasks belong to the nursing profession does justice to nursing as a profession. For instance, Abbott states in his contribution to the sociology of professions that each profession has autonomy and control over its work [47]. It was also expected that this would be reflected in the competency frameworks for RNs as this is the place to describe task divisions. However, it is not always clear how the jurisdiction (professional control) over tasks is divided between professions, both legally and in practice. For instance, the jurisdiction in prescribing medicine, a task originally belonging to the domain of the physician, varies greatly between countries, from RNs sharing jurisdiction to RNs being in a subordinate position [48]. Moreover, previous research has shown that task shifts from physicians to nurses are increasing and may lead to lower costs and similar or even better patient satisfaction and health outcomes [49–51]. As task shifting may be a solution for the shortages of healthcare workers, the WHO has recommended this as a strategy for countries to strengthen health systems [52, 53] and the European Union is looking for ways to implement this [54, 55]. In Western countries, specialized RNs and nurses with Master’s degrees in Advanced Nursing Practice are increasingly being allowed to take over tasks that belong to the domain of the physician, like prescribing medicine and diagnosing patients [56, 57]. In her research, Maier describes the considerable variation in the legal authority and control over these tasks, e.g. in who has prescription authority (nurses or physicians) [57].

The fact that our document analysis concerned ‘generic’ professional competency frameworks that transcend care settings and patient groups may partly explain why so little attention is paid to registered nurses’ specific tasks and why they are often described in imprecise terms. It is expected that more specific information on task division will be found in, for instance, competency frameworks for RNs specialized in the care for a specific patient group or nurses with a Master’s degree in Advanced Nursing Practice since they may have a more specific role within the nursing staff and also in relation to physicians.

### Limitations and strengths

A limitation of this document analysis concerns the selection of the five countries. Although a deliberate decision was made to include these specific countries, this does have consequences for the generalizability of the findings to the competency frameworks for RNs in other countries. For future research, it is recommended to extend this document analysis to include the professional competency frameworks of more countries.

Another limitation is that only the latest versions of the professional competency frameworks were included. As a result, we have not been able to identify possible changes over time. Some countries, like the Netherlands and Belgium, are currently revising the professional competency frameworks for RNs. When new frameworks are published, it would be interesting to compare them with the current version to see how the COVID-19 pandemic, for example, has affected the required competencies and tasks, e.g., in relation to the increasing use of e-health and online communication between RNs and patients.

The last limitation is that we only addressed the required competencies and task divisions as described in the competency frameworks. This research did not examine whether the framework is implemented in practice or is legally binding. For instance, in the Netherlands the professional competency framework is published but has not been anchored in the law or national regulations. Together with the variation in health systems between countries this could also be a possible explanation for the differences we found.

A strength of this document analysis is that it is the only known international study to date that compares current competency frameworks in different Western countries. This is especially interesting for nurses and nursing associations that develop new professional competency frameworks for RNs in collaboration with other stakeholders (e.g., governments and patient organizations). This analysis can also serve as input for the future alignment of competency frameworks between countries.

Another strength of this research is the involvement of experts from the respective countries, who were consulted both when retrieving the current competency frameworks and during the validation of the results for their respective countries.

## Conclusions

Ten generic domains were identified during the analysis and comparison of the professional competency frameworks. However, there are differences across countries in how the competencies of Bachelor-educated RNs are categorized and described in the frameworks. Moreover, limited information is available regarding the division of tasks between nurses, physicians and other healthcare professionals, particularly in Canada and the US. These variations and gaps in information may result in differences in nursing practice among countries and could impede the cross-border labour mobility for RNs.

### Electronic supplementary material

Below is the link to the electronic supplementary material.

Supplementary Material 1

## Data Availability

All professional competency frameworks can be found on the websites of the corresponding professional nursing associations as presented in the list below. Other data that support the findings of this study are available from the corresponding author upon reasonable request. Belgium. Professional and competency profile Nurse responsible for general care. Authorized by the Federal Council for Nursing (2016). Published by the Belgium Federal Public Service: Health, Food chain safety and Environment. https://overlegorganen.gezondheid.belgie.be/nl/documenten/beroeps-en-competentieprofiel-verpleegkundige-verantwoordelijk-voor-algemene-zorg (accessed: 19-04-2023). Canada. Entry-Level Competencies (ELCs) for the Practice of Registered Nurses (2018). Authorized and published by the Canadian Council of Registered Nurse Regulators (CCRNR). https://www.ccrnr.ca/entry-to-practice.html (accessed: 19-04-2023). The Netherlands. Toekomstbestendige beroepen in de verpleging en verzorging (2015). Developed in collaboration with the Nursing Association in the Netherlands (V&VN). https://www.venvn.nl/media/ctrip3kt/20160113-rapport-toekomstbestendige-beroepen.pdf (accessed: 19-04-2023). The United Kingdom. Future nurse: Standards of proficiency for registered nurses (2018). Authorized and published by The Nursing and Midwifery Council. https://www.nmc.org.uk/standards/standards-for-nurses/standards-of-proficiency-for-registered-nurses/ (accessed: 19-04-2023). The United States of America. The essentials: core competencies for professional nursing education (2021) Authorized and published by the American Association of Colleges of Nursing. https://www.aacnnursing.org/Essentials/Download-Order (accessed: 19-04-2023).
