# Supplementary material for: International comparison of professional competency frameworks for nurses: a document analysis
Source: BMC Nurs. 2023 Sep 28;22:343. doi: 10.1186/s12912-023-01514-3 (PMC10537821; doi:10.1186/s12912-023-01514-3)
Supplement: Supplementary file 1 — Supplementary Material 1 [file 12912_2023_1514_MOESM1_ESM.docx]

**Appendix 1**: Table of generic domains and corresponding competencies of registered nurses in the Netherlands, Belgium, United Kingdom, Canada and the United States.

| **Generic domain** | **Netherlands** | **Belgium** | **United Kingdom** | **Canada** | **United States** |
| --- | --- | --- | --- | --- | --- |
| **Professional Attitude** | Heading 4: Knowledge and Science: Reflective professional (p.19) e.g.  Participate in (practice)research.  Develop through self-reflection.  Can receive feedback from colleagues and supervisors and integrate it into her actions. Can give feedback to colleagues and students on their actions and professional behavior.  Be aware of moral and ethical values and act professionally in accordance with these.  Be a role model for (future) nurses.  Heading 7: Professionality and Quality (pp.20-21) e.g.  Know law and regulations and keep with the nursing code.  Be a self-confident and assertive nurse. | Heading 4: Taking professional responsibility (pp.20-22) e.g.  Pursuing ethical professional practice.  Evaluating oneself.  Demonstrate professional accountability.  Complying with laws and regulations.  Encourage a culture of health at work  Exercise professional leadership | Heading 1: Being an accountable professional (pp.7-9) e.g.: acting in the best interests of people, putting them first and providing nursing care that is person-centered, safe and compassionate.  They act  professionally at all times and use their knowledge  and experience to make evidence-based decisions  about care.  clear and transparent processes to investigate professionals who fall short of our standards.  to ensure that standards remain contemporary and fit for purpose in order to protect the public  clarity to the public and the professions about the core knowledge and skills that they can expect every registered nurse to demonstrate | Heading 2: Professional (pp. 5-6) e.g.  Registered nurses are professionals who are committed to the health and well-being of clients. Registered nurses uphold the profession’s practice standards and ethics and are accountable to the public and the profession.  Demonstrate a professional presence.  Exercises professional judgement.  Identifies and addresses ethical (moral) issues.   Heading 9: Scholar (p.9)  Registered nurses are scholars who demonstrate a lifelong commitment to excellence in practice through critical inquiry, continuous learning, application of evidence to practice, and support of research activities. | Heading 9: Professionalism (pp. 49-52) e.g.  Demonstrate an ethical comportment in one’s practice reflective of nursing’s mission to society.  Employ participatory approach to nursing care.  Demonstrate **accountability** to the individual, society, and the profession.  Comply with relevant laws, policies, and regulations.  Demonstrate the professional identity of nursing.  Integrate diversity, equity, and inclusion as core to one’s professional identity.  Heading 1: Knowledge for Nursing Practice (pp. 27-28) e.g.  Integration, translation, and application of established and evolving disciplinary nursing knowledge and ways of knowing, as well as knowledge from other disciplines, including a foundation in liberal arts and natural and social sciences. |
| **Clinical Care in Practice** | Heading 1: Care provider (p.17) e.g.  Can gather information in various ways, both at the level of the person requiring care and beyond the level of the person requiring care, analyse, interpret and apply this information;  Can enter into a care relationship, based on trust, focused on results with care recipients and various target groups;  Can carry out reserved and risky actions, taking into account his/her own competency and skills;  Competency 3.4. Is aware of chain processes and the organisation of healthcare in his/her own region; 3.5. Is familiar with (potential) cooperation partners outside of healthcare | Heading 1: Independently make a nursing diagnosis using current theoretical and clinical knowledge for the necessary nursing care. Plan, organize, implement and evaluate nursing care (pp. 12-154) e.g.  1.1. Making a nursing diagnosis independently using evidence based assessment techniques and instruments to gather the necessary data and arrive at a nursing diagnosis. Takes into account the holistic human view when collecting the data  1.3. Carrying out nursing care by applying capacity for critical thinking and uses a systematic approach to problem solving and nursing decision making in a wide range of professional contexts and in the delivery of care | Heading 4: Providing and evaluating care (pp. 16-18) e.g.: Providing evidence-based, compassionate and safe nursing interventions. They ensure that care they provide and delegate is person-centred and of a consistently high standard. They support people of all ages in a range of care settings. They work in partnership with people, families and carers to evaluate whether care is effective and the goals of care have been met in line with their wishes, preferences and desired outcomes.  Demonstrate the knowledge and ability to respond proactively and promptly to signs of deterioration or distress in mental, physical, cognitive and behavioural health and use this knowledge to make sound clinical decisions  Registered nurses in all fields of practice must demonstrate the  ability to provide nursing intervention and support for people of all ages who require nursing procedures during the processes of assessment, diagnosis, care and treatment for mental, physical,  cognitive and behavioural health challenges | Heading 1: Clinician (p.4) e.g.  Registered nurses are clinicians who provide safe, competent, ethical, compassionate, and evidence-informed care across the lifespan in response to client needs. Registered nurses integrate knowledge, skills, judgment and professional values from nursing and other diverse sources into their practice  Prepares clients for and performs procedures, treatments, and follow up care.  Applies knowledge of pharmacology and principles of safe medication practice.  Implements evidence-informed practices of pain prevention, manages client’s pain, and provides comfort through pharmacological and non-pharmacological interventions.  Implements therapeutic nursing interventions that contribute to the care and needs of the client. | Heading 2: Person-Centered Care (pp. 29-32) e.g.  Engage with the individual in establishing a caring relationship.  Integrate assessment skills in practice.  Diagnose actual or potential health problems and needs.  Demonstrate accountability for care delivery  Spheres of care: focuses on the individual within multiple complicated contexts, including family and/or important others.  Regenerative or restorative care; and hospice/palliative/supportive care are mentioned in the spheres of care (p.19) |
| **Communication and Collaboration** | Heading 2: Communicator (p.18) e.g.  Communicate at content, procedure and process level, and can empathize with patient. Use interview techniques at the level of the patient and is aware of (non)verbal and digital expressions. RNs are respectful and proficient in communicating with patients and their network and other professionals.  Heading 3: Collaborator (p.18) e.g.  RNs collaborate with patients, their network and other professionals, and support and refer patients. They can coordinate within a multidisciplinary team and position themselves when working together and take into account other visions. Efficiently and effective (digital) reporting and coach colleagues. | Heading 2: Communicating professionally with clarity (pp. 16-17) e.g.  Communicating professionally: active listening, emphasizing, respect patient opinion, respect other visions and confidentiality of care.  Inform patient to promote independence.  Report and share information: communicating orally, in writing or digitally coherent information.  Using information technology in the care process.  Heading 3: Cooperation (pp. 18-19) e.g.  Working together with the patient/client, their family and network, and with the interdisciplinary team  Competency 4.5: Builds a culture of collegiality, respect and professional relationships (p.21) | Heading 5: Leading and managing nursing care and working in teams (pp.19-20) e.g.  They play an active and equal role in the interdisciplinary team, collaborating and communicating effectively with a range of colleagues.  Heading 7: Coordinating care (pp.24-26) e.g. RNs understand and apply the principles of partnership,  collaboration and interagency working across all  relevant sectors  Competency 1.11: Communicate effectively using a range of skills and strategies with colleagues and people at all stage of life. (p.8)  Competency 2.9: use appropriate communication skills and strength based approaches to support and enable people to make informed choices. (p.12)  Competency 6.12: Understand the role of registered nurses and other health and care professionals at different levels of experience and seniority when managing and prioritising actions and care in the event of a major incident (p. 23) | Heading 3: Communicator (p.6) e.g.  Registered nurses are communicators who use a variety of strategies and relevant technologies to create and maintain professional relationships, share information, and foster therapeutic environments. Engages in active listening and communicate effectively in complex and rapidly changing situations and documents and reports clearly.  Heading 4: Collaborator (pp.6-7) e.g.  Registered nurses are collaborators who play an integral role in the health care team partnership. They initiate collaboration and determine their own professional and interprofessional role. | Heading 6: Interprofessional Partnerships (pp. 42-43)  Are e.g. intentional collaborations across professions and with care team members, patients, families, communities, and other stakeholders to optimize care, enhance the healthcare experience, and strengthen outcomes.  Communicate in a manner that facilitates a partnership approach to quality care delivery.  Perform effectively in different team roles using principles and values of team dynamics,  Use knowledge of nursing and other professions to address healthcare needs.  Work with other professions to maintain a climate of mutual learning, respect, and shared values.  Competency 2.2: communicate effectively with individuals. (p.29)  Competency 2.9c: Promote collaboration by clarifying responsibilities among individual, family, and team members (p. 32)  Competency 3.2: Engage in effective partnerships (p.34) |
| **Health Promotion and Prevention** | Heading 5: Health Promotor (pp.19-20) e.g.  Collect data in a broad contact aimed at early detection and risk assessment.  Can carry out interventions in individual and collective prevention and health education.  Has skills in outreach and mediation;  Is able to strengthen the social network around the person requiring care;  Takes into account personal factors, wishes and needs of groups of people, users and their relatives;  Can think along with policy makers and make proposals for necessary programs;  Can give reasoned advice on desired changes in lifestyle or maintenance of therapy | Heading 5: Empowering individuals, families and groups to adopt healthy lifestyles and care for themselves (p. 23) e.g.  Promote patient/client health and environment, incl. providing information to patient/client.  Learning patient/client about behaviour change strategies and evaluating this. | Heading 2: Promoting health and preventing ill health (pp.10-12) e.g.  Improving and maintaining the mental, physical and behavioural health and well-being of people, families, communities and populations. Support and enable people at all stage of life in all care settings to make informed decisions to manage health challenges and maximize quality of life and improve health outcomes. Be involved in prevention of ill health and engage in global health agendas and the reduction of health inequalities  Understand and apply the aims and principles of health  promotion, protection and improvement and the prevention of ill health when engaging with people. | Heading 7: Advocate (p.8) e.g.  Registered nurses are advocates who support clients to voice their needs to achieve optimal health outcomes. Registered nurses also support clients who cannot advocate for themselves.  Uses knowledge of population health, determinants of health, primary health care, and health promotion to achieve health equity.   Uses knowledge of health disparities and inequities to optimize health outcomes for all clients.  Competency 1.25: Uses strategies to promote wellness, to prevent illness, and to minimize disease and injury in clients, self, and others. (p.5)  Competency 1.27: Implements evidence-informed practices for infection prevention and control. (p. 5) | Heading 3: Population Health (pp.33-35) spans the healthcare delivery continuum from public health prevention to disease management of populations and describes collaborative activities with partnerships from affected communities, public health,  industry, academia, health care, local government entities, and others for the improvement  of equitable population health outcomes.  Competency 2.2e: Use evidence-based patient teaching materials, considering health literacy, vision, hearing, and cultural sensitivity. (p.30)  Competency 2.8c: Educate individuals and families regarding self-care for health promotion, illness prevention, and illness management. (p. 32)  Four Spheres of Care: 1) disease prevention/ promotion of health and well-being, which includes the promotion of physical and mental health in all patients as well as management of minor acute and intermittent care needs of generally healthy patients; 2) chronic disease care, which includes management of chronic diseases and prevention of negative sequelae; (p. 6) |
| **Organization and Planning of Care** | Heading 6: Organiser (p.20) e.g.   Is able to coordinate care around the patient, between disciplines and organisations, and to guarantee continuity of care, in consultation with the patient;  Is able to take decisions about policy (prioritising) and resources for individual patient care, whereby effect and costs are weighed up;  Handles materials and means responsibly;  Observes (behavioural) rules and protocols that belong to the professional responsibility;  Is able to work with the electronic patient/client file as a basis for action and to make proposals for improvement;  Is competent in the field of information and communication technology and is open to innovations in this field;  Contributes to patient safety and the working and learning climate within the organisation. (p. 18)  Competency 1.15: Can draw up, evaluate and adjust a care (life) plan in complex care situations (p.17)  Competency 3.16: Can establish, develop and apply a social map and social support systems (p.19) | Heading 6: Managing the care process (pp. 24-25) e.g.  Use the available resources as efficiently as possible, both economically and environmentally.  Delegate care to the most appropriate health professional in an effective and safe way.  Exercising organisational leadership by supporting the plan, goals and actions to promote and monitor the progress of an individual healthcare provider or the healthcare organisation.  Competency 1.2: Planning nursing care; Uses critical thinking skills and clinical reasoning in the planning process. Plans in cooperation with other professionals and the patient and informs the patient. Goals are formulated SMART and the care plan is revised and analysed regularly. (pp.13-14) | Heading 3: Assessing needs and planning care (pp.13-15) e.g. Prioritise the needs of people when assessing and reviewing their mental, physical, cognitive, behavioural, social and spiritual needs. They use information obtained during assessments to identify the priorities and requirements for person-centred and evidence-based nursing interventions and support. They work in partnership with people to develop person-centred care plans that take into account their circumstances, characteristics and preferences.  Understand the mechanisms that can be used to influence  organisational change and public policy, demonstrating the  development of political awareness and skills  Underpinning knowledge and skills required for their  role in coordinating and leading and managing the complex needs of people across organisations and settings. | Heading 5: Coordinator (p.7) e.g.  Registered nurses coordinate point-of-care health service delivery with clients, the health care team, and other sectors to ensure continuous, safe care.  Supports clients to navigate health care systems and other service sectors to optimize health and well-being.  Competency 1.5: Develops plans of care using critical inquiry to support professional judgment and reasoned decision-making. (p. 4)  Competency 6.5: Recognizes the impact of organizational culture and acts to enhance the quality of a professional and safe practice environment. (p. 7)  Competency 6.8: Uses and allocates resources wisely. (p. 8) | Heading 7: Systems-Based Practice (pp.44-45): Responding to and leading within complex systems of health care. Nurses effectively and proactively coordinate resources to provide safe, quality, and equitable care to diverse populations. E.g.  Apply knowledge of systems to work effectively across the continuum of care.  Incorporate consideration of cost-effectiveness of care.  Optimize system effectiveness through application of innovation and evidence-based practice.  Competency 2.5: develop a plan of care, organize care based on mutual health goals. (p.31)  Competency 2.9: Provide care coordination. (p.32) |
| **Leadership** | *Not a separate heading.*  Competency 3.10: Can fulfil a coordinating/regional role within a multidisciplinary team (p.18)  Competency 7.4: Has knowledge of quality frameworks and professional and personal leadership (p.18)  Appendix 4, 7F: Exercise the profession confidently and assertively, to be ambassadors for the profession and to demonstrate professional and personal leadership and to address colleagues and prospective nurses on professional and unprofessional behaviour (p.62) | *Not a separate heading.*  Nursing leadership (takes initiative to coordinating care) (p.8)  Competency 4.6: Exercising professional leadership Demonstrate professional leadership by participating in activities aimed at guiding policy and health services and making services more accessible; Supporting the dissemination, implementation, evaluation and adjustment of best practice recommendations; Contributing to a positive image of the nursing profession and helps shape a clear professional identity. (p.22)  Competency 6.3 Organizational leadership: Oversees the nursing care provided by others and remains accountable for the quality of care provided to care recipients. Supports the approach, goals and plans to facilitate and monitor the progress of an individual caregiver or the improvement of a care organisation. (p.25) | Heading 5: Leading and managing nursing care and working in teams (pp.19-20) e.g.  Registered nurses play a leadership role in coordinating  and managing the complex nursing and integrated care  needs of people at any stage of their lives, across a  range of organisations and settings. They contribute  to processes of organisational change through an  awareness of local and national policies.  Registered nurses provide leadership by acting as a  role model for best practice in the delivery of nursing  care.  understand the principles of effective leadership,  management, group and organisational dynamics and culture and apply these to team working and decision-making | Heading 6. Leader (pp.7-8) e.g.  Registered nurses are leaders who influence and inspire others to achieve optimal health outcomes for all.  Acts to enhance the quality of a professional and safe practice environment.  Demonstrates self-awareness. | Heading 10: Personal, Professional, and Leadership Development. (pp.53-54) e.g. Participation in activities and self-reflection that foster personal health, resilience, and well-being; contribute to lifelong learning; and support the acquisition of nursing expertise and the assertion of leadership.  Demonstrate a commitment to personal health and well-being.  Demonstrate a spirit of inquiry that fosters flexibility and professional maturity.  Develop capacity for leadership. |
| **Quality and Safety of Care** | Heading 7: Professional and quality promotor (p.20) e.g.  Initiate and develop matters in the area of quality assurance and innovation. Working in a result-oriented, effective and efficient way, complies with the nursing professional code, organisational rules and legislation.  Competency 1.18: In complex situations, can assess risks, identify problems early, choose and implement interventions, monitor progress and evaluate outcomes of care problems focused on the six dimensions of health (p. 17)  Competency 6.11: Contributes to patient safety and the working and learning climate within the organisation. (p.20) | Heading 7: Analyse, evaluate and ensure the quality of care provision in order to improve one's own practice  Basing one's practice on empirical, factual data; Argues for professional judgement based on knowledge of different sources, compelling evidence, the context and choice of care recipient.  Evaluating and ensuring quality; document and identify aspects nursing care and evaluate the quality, safety and effectiveness. (pp.25-27)  Competency 1.4: Evaluating nursing care; evaluate, report, document and share the results of nursing care in an accurate and structured way. (p.15)  Competency 4.3 Demonstrably taking professional responsibility: Strives to continuously improve the quality of his/her professional practice and the safety of patients/clients. Identifies and analyses incidents. (p.21) | Heading 6: Improving safety and quality of care (pp. 21-23) e.g.  Registered nurses make a key contribution to the continuous monitoring and quality improvement of care and treatment in order to enhance health outcomes and people’s experience of nursing and related care. They assess risks to safety or experience and take appropriate action to manage those, putting the best interests, needs and preferences of people first.  Demonstrate the ability to accurately undertake risk assessments in a range of care settings, using a range of contemporary assessment and improvement tools.  understand how the quality and effectiveness of nursing care can be evaluated in practice, and demonstrate how to use service delivery evaluation and audit findings to bring about continuous improvement  Competency 3.16: Demonstrate knowledge of when and how to refer people  safely to other professionals or services for clinical intervention or support. (p.15) | *Not a separate heading.*  Competency 1.1: Provides safe, ethical, competent, compassionate, client-centred and evidence-informed nursing care across the lifespan in response to client needs. (p.4)  Competency 1.8: Recognizes and responds immediately when client safety is affected. (p.4)  Competency 1.16: Incorporates principles of harm reduction with respect to substance use and misuse into plans of care. (p.4)  Competency 2.13: Recognizes, acts on, and reports, harmful incidences, near misses, and no harm incidences. (p.6)  Competency 2.14: Recognizes, acts on, and reports actual and potential workplace and occupational safety risks. (p.6)  Competency 7.1: Recognizes and takes action in situations where client safety is actually or potentially compromised. (p.8)  Competency 7.6: Advocates for safe, competent, compassionate and ethical care for clients. (p.8)  Competency 7.10: Advocates for client’s rights and ensures informed consent, guided by legislation, practice standards, and ethics.(p.8) | Heading 5: Quality and Safety (pp.39-41) is the employment of established and emerging principles of safety and improvement science. Quality and safety, as core values of nursing practice, enhance quality and minimize risk of harm to patients and providers through both system effectiveness and  individual performance.  Apply quality improvement principles in care delivery by recognizing nursing’s essential role in improving healthcare and safety and compare quality improvement methods and develop a plan of monitoring quality and improvement change.  Contribute to a culture of patient safety: Examine basic safety design principles to reduce risk of harm. Contribute to a culture of provider and work environment safety by identifying actual and potential level of risks to providers within the workplace.  Competency 2.5d: Incorporate evidence-based intervention to improve outcomes and safety. (p.31)  Competency 8.3e: Identify impact of information and communication technology on quality and safety of care. 47 |
| **Training and (continuing) Education** | *Not a separate heading.*  Competency 3.17: Can supervise and coach colleague care providers; (student) higher professional education nurses, senior secondary vocational education nurses, carers, etc. (p.19)  Competency 4.9: Keeps up with professional literature. Knows how to adequately look up information and assess its value (p.19)  Competency 4.11: Can develop himself through self-reflection and self-assessment of his own results.  Competency 4.13: Can receive feedback from colleagues and supervisors and integrate it into her actions. (p.19)  Competency 4.14: Can give feedback to colleagues and students on their actions and professional conduct.(p.19)  Competency 4.18: Is able to act as a role model for (prospective) nurses. (p.19)  Competency 7.14: Can indicate the limits of her competency and skills to colleagues and clients. (p.21) | *Not a separate heading.*  Competency 4.2: Evaluates himself in order to identify strengths and areas for improvement. Interacts with colleagues to further develop his/her professional practice. (p.20)  Competency 4.3: Continuously maintains and sharpens his/her competencies. Participates in further training. (p.21)  Competency 7.1: Participates in research and formulates recommendations for best practices. (p.25)  Competency 7.3: Participates in the education of students and colleagues to develop the nursing profession and the quality of health care. Contributing to the education of students and health professionals by being role model and reference for students, colleagues and other professionals. Pay attention to the questions and problems of students and colleagues. Guides students and colleagues in analysing their practice. Provides formal or informal constructive feedback in the context of assessing students and colleagues. Encourages independent learning and searching for solutions. (pp.26-27) | *Not a separate heading.*  Competency 1.17: take responsibility for continuous self-reflection, seeking and responding to support and feedback to develop their professional knowledge and skills. (p8)  Competency 5.8: support and supervise students in the delivery of nursing care, promoting reflection and providing constructive feedback, and evaluating and documenting their performance. (p.20)  Competency 5.9: demonstrate the ability to challenge and provide constructive feedback about care delivered by others in the team, and support them to identify and agree individual learning needs. (p.20) | Heading 8: Educator (p.9) e.g.  Registered nurses are educators who identify learning needs with clients and apply a broad range of educational strategies towards achieving optimal health outcomes  Selects, develops, and uses relevant teaching and learning theories and strategies to address diverse clients and contexts, including lifespan, family, and cultural considerations.  Heading 9: Scholar (p.9) e.g.  Registered nurses are scholars who demonstrate a lifelong commitment to excellence in practice through critical inquiry, continuous learning, application of evidence to practice, and support of research activities.  Supports research activities and develops own research skills.  Engages in practices that contribute to lifelong learning | Heading 4: Scholarship for Nursing Discipline (pp.37-38) is the generation, synthesis, translation, application, and dissemination of nursing  knowledge to improve health and transform health care.e.g.  Participate in scholarly inquiry as a team member.  Demonstrate ethical behaviors in scholarly projects including quality improvement and EBP initiatives.  Competency 2.8c: Educate individuals and families regarding self-care for health promotion, illness prevention, and illness management. (p.32)  Competency 9.3h: Engage in peer evaluation. (p.50)  Competency 10a-e: a.Engage in guided and spontaneous reflection of one’s practice. b Integrate comprehensive feedback to improve performance. c Commit to personal and professional development. d Expand personal knowledge to inform clinical judgment. e Identify role models and mentors to support professional growth.(p.53) |
| **Technology and e-health** | *Not a separate heading.*  Competency 2.17: Is digitally literate, at home on the Internet and makes professional use of e-health, remote care and social media (p.18)  Competency 3.13: Is able to efficiently and effectively (digitally) report, discuss and transfer (also across organisation boundaries) (p.19)  Competency 6.9: Is able to work with the electronic patient/client file as a basis for action and to make proposals for improvement. (p.20)  Competency 6.10: Is competent in the field of information and communication technology and is open to innovations in this field. (p.20)  Competency 7.7: Is able to initiate and develop matters in the field of quality care and innovation (including  ICT). (p.20) | *Not a separate heading.*  Competency 1.3: Uses available technology and ICT to store, access and record data and other (nursing) information about the patient/client.  Uses technologies available in the health care environment to maximise access to health care and optimise patient/client outcomes. (pp.14-15)  Competency 2.3: Communicates clear, coherent information orally, in writing or electronically in an accurate manner, using professional terms. (p.17)  Competency 2.4: Uses information technology in the context of the care process; uses and masters the tools imposed by the government. Is familiar with IT architecture. Manages the nursing part of an electronic patient file. (p.17) | *Not a separate heading.*  Competency 1.15: Demonstrate the numeracy, literacy, digital and technological skills required to meet the needs of people in their care to ensure safe and effective nursing practice. (p.9)  Competency 5.11: Effectively and responsibly use a range of digital technologies  to access, input, share and apply information and data within  teams and between agencies. (p.20)  Annex B. 2.2: Take, record and interpret vital signs manually and via technological devices. (p.32) | *Not a separate heading.*  Competency 2.8: Demonstrates professional judgment to ensure social media and information and communication technologies (ICTs) are used in a way that maintains public trust in the profession. (p.5)  Competency 3.6: Uses information and communication technologies (ICTs) to support communication. (p.6)  Competency 8.5: Assists clients to access, review, and evaluate information they retrieve using information and communication technologies (ICTs). (p.9)  Competency 9.4: Engages in activities to strengthen competency in nursing informatics. (p.9)  Competency 9.5: Identifies and analyzes emerging evidence and technologies that may change, enhance, or support health care. (p.9) | Heading 8: Informatics and Healthcare Technologies (pp.46-48) are used to  provide care, gather data, form information to drive decision making, and support professionals as they expand knowledge and wisdom for practice. Informatics processes and technologies are used to manage and improve the delivery of safe, high-quality, and efficient healthcare services in accordance with best practice and professional and regulatory standards.  Describe the various information and communication technology tools used in the care of patients, communities, and populations.  Use information and communication technology to gather data, create information, and generate knowledge. And to support documentation of care and communication among providers, patients, and all system levels.  Use information and communication technologies and informatics processes to deliver safe nursing care to diverse populations in a variety of settings.  Use information and communication technologies in accordance with ethical, legal, professional, and regulatory standards, and workplace policies in the delivery of care. |
| **Support of Self-management and Patient Empowerment** | *Not a separate heading.*  The RNs are professionals who focus on promoting and supporting the self-management of care recipients, their relatives and their social network, with the objective of maintaining or improving their functioning in relation to quality of life, health and disease (p.12)  Competency 1.7: Is able to determine, based on clinical reasoning, the nursing care aimed at maintaining or (re)acquiring the self-management of the caretaker and his/her relatives (p.17)  Competency 1.10: Is able to support and promote the self-management of caretakers, their relatives and their social network with the aim of maintaining or improving the functioning in relation to quality of life, health and illness (p.17) | *Not a separate heading.*  In providing this care, the nurse focuses on the patient's/client's total experience and takes a holistic view. The focus is primarily on advocacy and empowerment of the patient/client (p.5)  The patient/client is the most important partner in this partnership. The emphasis is on the self-management and empowerment of the patient/client, but account is also taken of the specific care relationship in which the patient/client is or becomes more or less dependent on the nurse. Then advocacy is needed (p.7)  Competency 1.3: Encourages the self-reliance of the patient/client to maximise the level of self-management and quality of life (p.15)  Competency 3.1: Encourages patient/client self-determination in making health choices (p.18) | *Not a separate heading.*  Empowering people, communities and populations to exercise choice, take control of their own health decisions and behaviours, and by supporting people to manage their own care where possible (p. 3)  Competency 4.2: work in partnership with people to encourage shared decision making in order to support individuals, their families and carers to manage their own care when appropriate (p.17)  Competency 4.3: demonstrate the knowledge, communication and relationship management skills required to provide people, families and carers with accurate information that meets their needs before, during and after a range of intervention (p.17)  Annex B. 3.: Accurately assessing the person’s  capacity for independence and self-care and initiating  appropriate interventions (p.33) | *Not a separate heading.*  Competency 5.6: Supports clients to navigate health care systems and other service sectors to optimize health and well-being. (p.7)  Competency 7.7: Support and empower clients in making informed decisions about their health care, and respects their decisions. (p.8) | *Not a separate heading.*  Competency 2.8: Promote self-care management. Assist the patient to engage in self-care management. Educate regarding self-care of illness management. Respect self-determination in their healthcare decisions. Identify personal, system and community resources available to support self-care management. (p.32) |
